# Review of the Arbitrium (ARM) System: Molecular Mechanisms, Ecological Impacts, and Applications in Phage–Host Communication

**DOI:** 10.3390/microorganisms13092058

**Published:** 2025-09-04

**Authors:** Junjie Shang, Qian Zhou, Yunlin Wei

**Affiliations:** Faculty of Life Science and Technology, Kunming University of Science and Technology, Kunming 650500, China; sjjnjy117327@gmail.com (J.S.); 17687026368@163.com (Q.Z.)

**Keywords:** bacteriophages, arbitrium system, quorum-sensing, phage–host communication

## Abstract

Bacteriophages (phages) play a pivotal role in shaping microbial communities and driving bacterial evolution. Among the diverse mechanisms governing phage–host interactions, the Arbitrium (ARM) communication system represents a recently discovered paradigm in phage decision-making between the lytic and lysogenic cycles. Initially identified in *Bacillus*-infecting phages, the ARM system employs a quorum-sensing-like peptide signaling mechanism to modulate infection dynamics and optimize population-level survival strategies. Recent studies have elucidated the structural and functional basis of ARM regulation, highlighting its potential applications in antimicrobial therapy, microbiome engineering, and synthetic biology. The significance of ARM systems lies in their ability to regulate bacterial population stability and influence the evolutionary trajectories of microbial ecosystems. Despite being a relatively recent discovery, ARM systems have garnered considerable attention due to their role in decoding phage population dynamics at the molecular level and their promising biotechnological applications. This review synthesizes current advancements in understanding ARM systems, including their molecular mechanisms, ecological implications, and translational potential. By integrating recent findings, we provide a comprehensive framework to guide future research on phage–host communication and its potential for innovative therapeutic strategies.

## 1. Introduction

Temperate bacteriophages employ diverse strategies to regulate their life cycle, balancing lytic replication with lysogenic integration [1]. Among these regulatory mechanisms, the ARM communication system, first identified in *Bacillus*-infecting phages, represents a paradigm-shifting model of phage decision-making [2]. In this system, small peptides such as AimP, secreted during infection, function as quorum-sensing signals that enable phages to coordinate population-level transitions between lysis and lysogeny [2]. Beyond individual infections, ARM systems play a crucial role in shaping microbial ecosystems. By dynamically modulating the lytic–lysogenic switch in response to host density, ARM-regulated phages contribute to bacterial population stability, facilitate horizontal gene transfer, and enhance ecosystem resilience—factors with significant implications for environmental microbiology and human microbiome management [3].

The resurgence of phage therapy as a solution to antibiotic resistance highlights the need for precise control over phage lifecycles. ARM systems, with their peptide-based signaling and modular design, offer unprecedented opportunities to engineer phages for targeted pathogen clearance while preserving commensal microbiota. This review synthesizes recent advances in ARM system biology, explores their ecological and evolutionary implications, and evaluates their translational potential in phage therapy and microbiome engineering.

## 2. Molecular Mechanisms of ARM Systems

### 2.1. The AimP-AimR-AimX Signal Transduction Pathway

The ARM system regulates the lysis-lysogeny decision through a finely tuned signal transduction pathway involving three key components: the signaling peptide AimP, its receptor AimR, and the transcriptional regulator AimX. This pathway allows phages to dynamically sense and respond to environmental cues, particularly host density, by modulating transcriptional programs that determine viral fate [4].

Signal Production and Secretion: AimP as a Quorum-Sensing Peptide: During phage infection, the *AimP* gene is transcribed and translated into a precursor peptide. In Bacillus phages, AimP is initially synthesized as a 30-amino acid propeptide containing an N-terminal secretion signal and a C-terminal inhibitory domain [2]. Host-encoded proteases subsequently cleave the precursor, generating the mature AimP peptide, which typically comprises 6 residues [5]. The mature AimP peptide is then secreted into the extracellular milieu via the host’s Sec or Tat secretion systems [6], with its concentration directly reflecting the density of infected host cells. This accumulation functions as a quorum-sensing signal, enabling phages to regulate infection dynamics in response to population density [2,6].

**Figure 1:**
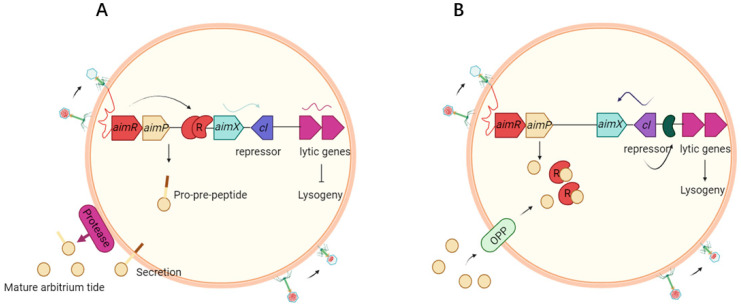
Mechanistic model for lysis-lysogeny decisions. (**A**) At the first encounter of a phage with a bacterial population, AimR dimer binds *AimX* promoter via HTH motifs. AimR activates AimX expression, AimX transcription initiates, promoting lytic gene expression. Phage replicates and lyses host, at the same time AimP is expressed, secreted and processed extracellularly to produce the mature peptide. (**B**) At later stages of the infection dynamics, the arbitrium peptide accumulates in the medium. AimP binds AimR TPR domain, inducing conformational closure. HTH motifs detach from DNA, silencing *AimX*. Lysogeny maintenance genes (e.g., *cI*) dominate, enabling genome integration (figure created in BioRender.com).

Signal Detection and Conformational Switching: AimR as the Receptor: AimR functions as a homodimeric transcription factor composed of two key domains: a tetratricopeptide repeat (TPR) domain that mediates AimP binding and a helix-turn-helix (HTH) motif responsible for recognizing the AimX promoter region [7]. During the lytic phase, when host density is low and AimP is absent, AimR dimers adopt an “open” conformation, allowing the HTH domain to bind the AimX promoter and activate transcription [7] (Figure 1A). In contrast, under high host density conditions associated with the lysogenic phase, AimP accumulates inside the bacterial cell (via Oligopeptide Permease (Opp) transporters) and binds to the TPR domain of AimR. This binding induces a significant conformational change in AimR to a “closed” state, which sterically hinders the HTH motif from accessing DNA, effectively repressing AimX transcription [2,7,8] (Figure 1B). X-ray crystallographic studies have provided detailed insights into these conformational transitions, underscoring the modularity and adaptability of AimR in regulating phage decision-making processes [7,8].

Transcriptional Output: the transcriptional output of this pathway is governed by AimX, which encodes either a small regulatory RNA or a protein that acts as the master switch determining phage fate [2,9]. During the lytic cycle, AimX facilitates the expression of phage lytic genes (e.g., holins, endolysins, nucleases, lysin A, phage tail proteins) while concurrently repressing factors that sustain lysogeny [2,9]. By orchestrating this multifaceted lysis process, AimX ensures rapid bacterial cell death and efficient release of new phage particles [7]. Conversely, during the lysogenic cycle, suppression of AimX permits the dominance of integrases and repressors (e.g., CI-like proteins), thereby promoting the integration of the phage genome into the host chromosome [10].

Environmental Modulation by Host Density: The structural switch in AimR translates extracellular host density (via AimP concentration) into the transcriptional fate of AimX and thus the phage lifecycle. At high bacterial densities, elevated AimP levels saturate the TPR domain of AimR, inducing the conformational closure that prevents DNA binding and represses AimX expression [2,7]. This repression facilitates the transition to and maintenance of the lysogenic cycle, allowing the phage genome to integrate and persist within a stable host population, mitigating excessive host depletion [10,11]. Conversely, at low bacterial densities, reduced AimP concentrations fail to inhibit AimR activity. The “open” conformation of AimR allows HTH binding and activation of AimX transcription [12]. This shift promotes the lytic cycle, ensuring rapid production of new phage progeny to exploit scarce hosts and maximize propagation [13]. Through this dynamic switching mechanism, phages optimize their survival strategy—integrating during host abundance and lysing during scarcity [14].

### 2.2. Structural Plasticity and Environmental Responsiveness

The structural basis of the AimR conformational switch provides the ARM system with remarkable plasticity and responsiveness to environmental fluctuations, particularly host density. As described above, the binding of the small peptide AimP to the TPR domain triggers a profound allosteric change in AimR, repositioning its HTH domain away from the DNA binding site [7,8]. This modular design—where a sensory domain (TPR) controls the activity of an effector domain (HTH)—is reminiscent of eukaryotic signal transduction pathways and offers significant evolutionary flexibility [15].

This structural plasticity underpins the system’s ability to finely tune the lysis-lysogeny decision in real-time based on the concentration of the diffusible AimP signal, which serves as a direct readout of infected host density in the local environment [2,12]. The sensitivity of this switch allows phages to rapidly adapt their reproductive strategy to optimize survival and propagation across diverse and dynamic ecological niches, such as the heterogeneous soil matrix, aquatic environments with seasonal blooms, or the competitive human microbiome [3,13,16].

### 2.3. Implications for Synthetic Biology

The modular nature and peptide-based signaling of the AimP-AimR-AimX pathway offer unprecedented opportunities for bioengineering applications, particularly in synthetic biology and phage therapy [17,18]. The clear separation of the sensory module (AimP detection by AimR TPR) from the effector module (AimR HTH controlling AimX) provides a plug-and-play framework.

Engineering Lifecycle Control: Targeted mutations in AimR residues critical for AimP binding or conformational change can decouple the signaling mechanism from natural host density cues [19]. This enables the design of phages with constitutively active (always lytic) or externally inducible (e.g., by synthetic analogs or specific environmental triggers) lifecycle switches, offering precise spatiotemporal control over therapeutic phage activity [11,19].

Advantages Over Classic Systems: This programmability contrasts with the complexity of engineering classic systems like the lambda cI/Cro switch. Lambda’s feedback mechanism relies on intricate protein-DNA interactions and competitive binding of multiple regulators to overlapping operator sites, making it less amenable to straightforward re-wiring for novel inputs or outputs [14,20,21].

Smart Antimicrobials and Beyond: Engineered ARM phages could be programmed to sense not only bacterial density but also specific disease-relevant metabolites or biomarkers within the host environment, triggering lysis only under predefined conditions [18,19]. This enhances therapeutic precision, minimizes off-target effects on commensal microbiota, and reduces the likelihood of resistance development by avoiding overexploitation. Furthermore, integrating ARM modules into synthetic microbial consortia could enable the creation of programmable communities for tasks like targeted drug delivery or environmental bioremediation, where population-level behaviors are dynamically regulated by designed communication signals [22].

Future research should prioritize elucidating the precise molecular function and direct targets of AimX (whether RNA or protein) and its interactions with host regulatory networks. Deeper understanding of these relationships will be crucial for fully harnessing the potential of ARM systems in therapeutic and biotechnological applications [19].

## 3. Comparative Analysis: ARM System vs. Classic Phage Regulatory Mechanisms

The lysis-lysogeny decision in temperate bacteriophages is regulated by diverse mechanisms, with the ARM system and the Lambda phage CI/Cro system representing two distinct paradigms. While the Lambda phage has long served as a model for phage lifecycle regulation [23], the recent discovery of ARM systems has unveiled novel regulatory strategies with unique advantages (Table 1).

### 3.1. Molecular Signaling and Environmental Adaptation

To contextualize the novelty and advantages of the ARM system, we compare its core features with the well-established lambda phage CI/Cro regulatory paradigm [14]. The ARM system and the Lambda phage CI/Cro system differ significantly in their molecular signaling pathways and environmental adaptability. The ARM system, composed of AimP, AimR, and AimX, is highly dynamic and responsive to environmental changes, particularly host bacterial density. It can finely regulate the lysis-lysogeny decision by using AimP as a signal molecule to modulate AimR activity. This allows phages to precisely adjust their life cycle, inducing lysis at low bacterial densities and lysogeny at high densities. This adaptability is a key advantage for applications in phage therapy and microbial community engineering, where environmental conditions often fluctuate [11]. Additionally, ARM’s modular nature offers the ability to engineer phages for specific responses to external cues, enhancing its potential for targeted antimicrobial treatment and synthetic biology applications.

In contrast, the Lambda CI/Cro system relies on a simpler regulatory mechanism where the balance between CI (which maintains lysogeny) and Cro (which promotes lysis) controls the phage’s life cycle [4,12]. However, Lambda’s response is slower and less sensitive to environmental factors such as bacterial density, limiting its adaptability in dynamic environments. While useful in stable conditions, the Lambda system lacks the flexibility and precision that ARM systems offer in more complex or variable settings.

Given its real-time response to environmental signals and programmable nature, the ARM system holds significant promise for synthetic biology and phage therapy, enabling more efficient and tailored approaches to managing bacterial infections and microbial communities. Future research should explore the full range of ARM system targets and its interactions with host metabolic networks, paving the way for more innovative antimicrobial solutions and microbial consortia engineering.

### 3.2. Structural and Evolutionary Insights

The structural and evolutionary characteristics of the ARM system present a striking contrast to Lambda phage’s regulatory system [5], particularly in terms of the interaction between protein domains and DNA. In ARM systems, the AimR protein features a tetratricopeptide repeat (TPR) domain that undergoes a significant conformational shift upon binding to AimP, the signaling peptide [20]. This structural rearrangement effectively prevents AimR from recognizing its DNA target, thus regulating gene expression in a manner similar to eukaryotic signal transduction pathways [6]. The ability to modulate DNA binding through conformational changes provides the ARM system with remarkable flexibility and dynamic adaptability, allowing it to respond rapidly to environmental cues, such as changes in host bacterial density [8].

In contrast, the Lambda phage relies on CI and Cro proteins, which function through helix-turn-helix (HTH) motifs to bind to overlapping operator sites on the DNA. This rigid, competition-based mechanism limits the evolutionary flexibility of Lambda’s regulatory system [4]. The HTH motifs lock the proteins into a more static DNA-binding conformation, which restricts their ability to adapt as easily to fluctuating environmental conditions. As a result, Lambda’s feedback system is less adaptable compared to the modular ARM system, where the molecular components can evolve and adapt more freely.

This difference in structural flexibility highlights the modularity of ARM systems, which may contribute significantly to their evolutionary success across various phage families. The dynamic conformational shift in AimR allows ARM systems to easily diverge and recombine within different phage lineages, fostering horizontal gene transfer and enabling phages to rapidly evolve and adapt to new host environments. This modular nature contrasts with the more rigid Lambda system, which faces greater constraints in terms of horizontal transfer potential and adaptability.

The structural flexibility conferred by the TPR domain’s conformational shift allows ARM systems greater evolutionary adaptability compared to the more rigid, HTH-based competitive binding mechanism of lambda [14,15]. This modularity facilitates horizontal gene transfer and may explain the widespread utilization of peptide communication (like AimP) observed across diverse phage families infecting various hosts, including soil and pathogenic bacteria [3,13,16]. The ability of phages to communicate through peptides, such as AimP in ARM systems, plays a crucial role in phage–host interactions, providing a versatile mechanism for fine-tuning the balance between lysis and lysogeny. Studies have shown that soil-infecting phages like *Bacillus* phages utilize peptide signaling to sense bacterial population density in the rhizosphere, promoting lysogeny in high-density zones and avoiding over-exploitation of bacterial hosts [14].

The peptide-based communication system seen in ARM systems is not only an effective strategy for maintaining host stability but also has implications for phage therapy [18,19]. The flexibility and adaptability of the ARM system allow it to be engineered for use in targeted antimicrobial applications, where phages can be programmed to respond dynamically to bacterial population shifts, enhancing therapeutic efficacy while minimizing the risk of resistance development [21]. The evolutionary advantage of such peptide communication in diverse environments, including soil and pathogenic bacteria, underscores its broad applicability across different ecological niches, highlighting the evolutionary flexibility of ARM systems compared to more rigid models like the Lambda phage CI/Cro system. These structural and evolutionary insights highlight the ARM system as a flexible regulatory paradigm, which raises important ecological and translational questions further explored in Section 4 and Section 5.

## 4. Ecological Impact of ARM Systems

### 4.1. Environmental Specificity of ARM-Mediated Regulation

The ecological impact of ARM systems is profoundly shaped by the distinct environmental pressures encountered in different habitats. The system’s responsiveness to host density allows phages to implement niche-specific strategies for survival and ecosystem influence.

#### 4.1.1. Soil Ecosystems: Lysogeny for Stability

Soil presents a nutrient-rich yet spatially and temporally heterogeneous environment. Fluctuations in bacterial density due to nutrient patches, root exudates, moisture gradients, and temperature variations strongly influence phage strategies [3,25,26]. ARM-regulated *Bacillus* phages (e.g., SPβ) utilize the accumulating AimP signal in high-density zones, such as the rhizosphere where *Bacillus subtilis* populations thrive, to induce lysogeny [3,27]. This strategy prevents overexploitation of the bacterial host reservoir, promoting population stability crucial for sustained microbial functions like organic matter decomposition and nutrient cycling—processes vital for soil health and plant growth [3,28]. Factors like soil pH (affecting nutrient solubility and microbial activity) [25] and moisture/temperature fluctuations [29] further modulate bacterial densities and activity, consequently influencing AimP accumulation and the ARM-mediated balance between lysis and lysogeny. Plant-microbe interactions (e.g., root exudates stimulating bacterial growth) and microbial competition also contribute to the complex dynamics shaping ARM functionality in soil [3].

#### 4.1.2. Aquatic Ecosystems: Balancing Lysis and Dispersal

**Figure 2:**
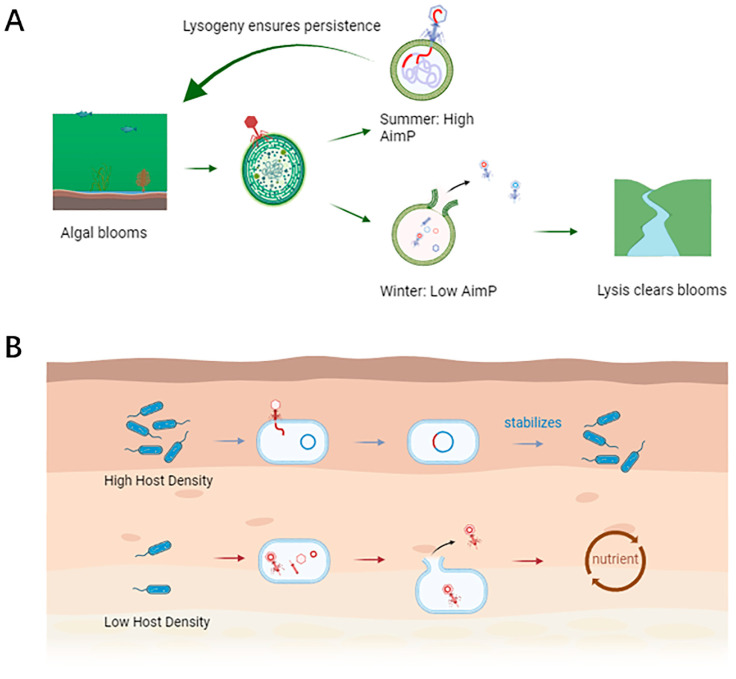
ARM system dynamics in aquatic and soil microbiomes. (**A**) Aquatic environment: with the shifts in seasons, in winter, the host is scarce, AimP is low, and phage lysis hosts clear water blooms; there are more hosts in summer, AimP accumulates, and the phage tends to lysogeny, so that the host population remains stable. (**B**) Soil Environment: under high host density, AimP accumulates, lysogeny dominates, phage integration stabilizes host populations; at low host density, AimP is less, phage lysis hosts and resource recycling (figure created in BioRender.com).

Aquatic environments (freshwater, marine) exhibit significant seasonal and spatial variations in bacterial host availability. ARM systems enable temperate phages to dynamically adapt. In periods of host scarcity (e.g., winter stratification in lakes), high phage densities lead to AimP accumulation, promoting lysogeny [13,30]. This allows prophage persistence within dormant hosts until conditions improve. Conversely, during transient surges in host abundance, such as phytoplankton blooms stimulating associated bacterial growth (e.g., *cyanobacteria*), the dilution of extracellular AimP favors the lytic cycle [30,31]. This rapid shift maximizes viral progeny production by exploiting the ephemeral abundance of susceptible hosts, facilitating dispersal and infection of new populations [13,30]. Figure 2 illustrates these contrasting dynamics.

### 4.2. Ecological Consequences of Lysogeny and Lysis Switching

Beyond optimizing phage survival, the ARM-mediated switch between lysis and lysogeny exerts profound and distinct ecological influences:

Lysogeny as an Ecological Stabilizer and Gene Reservoir: In environments like soil, lysogeny driven by high host density acts as a buffer against boom-bust cycles. It maintains a reservoir of dormant phages within bacterial genomes, enhancing bacterial survival under stress and enabling rapid community recovery when conditions stabilize [3,28]. Critically, lysogeny facilitates prophage-mediated horizontal gene transfer (HGT), disseminating traits like antibiotic resistance genes (ARGs) or metabolic capabilities across bacterial populations [32].

Lysis as a Population Control Mechanism: In aquatic systems, the ARM-triggered shift to lysis during bacterial blooms (e.g., fueled by nutrient influx) provides essential top-down control [31]. By rapidly lysing abundant hosts, phages prevent unchecked bacterial proliferation, thereby mitigating risks associated with eutrophication such as oxygen depletion, harmful algal blooms, and biodiversity loss [30,31,33]. This phage-mediated lysis also accelerates nutrient recycling, fueling microbial food webs [13,33].

### 4.3. Response to Anthropogenic Perturbations

Human activities are altering environments in ways that disrupt natural ARM-mediated balances, with significant ecological and public health implications:

Agricultural Intensification: Excessive fertilizer application elevates soil nutrient levels, boosting bacterial densities [25,32]. This favors ARM-driven lysogeny [3], inadvertently increasing prophage integration and HGT. Consequently, environments like fertilized agricultural soils become hotspots for the accumulation and dissemination of antibiotic resistance genes (ARGs) encoded on mobile genetic elements, including prophages [32,34]. This process potentially accelerates the emergence and spread of multidrug-resistant bacteria, exacerbating the global antibiotic resistance crisis [32].

Climate Change: Rising global temperatures destabilize microbial ecosystems [35]. While phages often favor lysogeny under stable conditions, increased temperatures can disrupt this equilibrium, promoting a shift towards the lytic cycle [13,35]. Enhanced bacterial lysis alters community composition, reduces biomass, and disrupts essential ecosystem functions like carbon sequestration and nutrient cycling [13,35]. In marine environments, this phage-mediated response could amplify the negative impacts of climate change on ocean health and productivity [13].

### 4.4. Ecological Impacts of ARM-Mediated Regulation

ARM-mediated regulation plays a crucial role in shaping microbial community dynamics, particularly through lysogeny and its ability to modulate bacterial behavior in response to environmental fluctuations. In soil ecosystems, ARM-driven lysogeny stabilizes bacterial populations by buffering against boom-bust cycles triggered by nutrient fluctuations or environmental stress [14]. By maintaining a reservoir of dormant phages within bacterial genomes, lysogeny enables bacterial survival under adverse conditions and facilitates the resumption of normal metabolic activity once environmental stability is restored. This regulatory mechanism is essential for sustaining nutrient cycling and organic matter decomposition, both of which are fundamental to soil health and overall ecosystem function. Without this balance, disruptions in nutrient cycling could negatively impact plant growth and soil fertility [15].

In aquatic ecosystems, ARM-regulated phages play a critical role in maintaining microbial balance by modulating bacterial population dynamics. During host blooms, often driven by excess nutrients such as nitrogen and phosphorus, phages that transition to the lytic cycle rapidly lyse their bacterial hosts, preventing unchecked bacterial proliferation [36]. This biological control mechanism helps mitigate the risk of eutrophication—a process in which excessive nutrient input leads to oxygen depletion, harmful algal blooms, and biodiversity loss. By inducing lytic bursts during these blooms, ARM phages contribute to ecological stability, ensuring bacterial populations remain regulated and preventing the cascading effects of nutrient overload [37].

### 4.5. Anthropogenic Perturbations and ARM Responses

Anthropogenic disturbances, such as intensive agricultural practices and climate change, profoundly impact microbial dynamics, influencing ARM-encoded phages in ways that affect both microbial communities and human health. For instance, excessive fertilizer use in agriculture significantly increases nutrient availability in soil, leading to higher bacterial densities [38]. This shift favors lysogeny, wherein phage genomes integrate into host DNA, promoting prophage-mediated horizontal gene transfer (HGT)—a process that facilitates the dissemination of antibiotic resistance genes (ARGs) among bacterial species. In the case of ARM phages, this mechanism may accelerate the spread of ARGs, potentially exacerbating the global antibiotic resistance crisis [16]. The increased lysogeny driven by elevated host densities in fertilized soils creates a reservoir of resistance genes that can be mobilized by environmental stressors, contributing to the rapid evolution of multidrug-resistant bacteria [16,27].

Similarly, climate change exerts significant pressure on microbial ecosystems [39]. Phages typically favor lysogeny under stable environmental conditions, but rising temperatures disrupt this balance, promoting a shift toward the lytic cycle. This transition increases bacterial cell lysis, reshaping microbial community composition and further destabilizing ecosystem function. In marine environments, such disruptions could exacerbate the negative effects of climate change on ocean health, underscoring the intricate ways in which human activities influence phage dynamics and microbial ecology [30].

ARM systems stabilize soil ecosystems through density-dependent lysogeny and enable aquatic phages to adapt to seasonal and spatial fluctuations, thereby influencing nutrient cycling and community dynamics [29,30]. Understanding these mechanisms is essential for predicting microbial responses to global change [28].

## 5. Application and Prospect

The ARM system has emerged as a pivotal framework for advancing phage therapy and microbiome engineering, offering unparalleled precision in regulating phage lifecycles and shaping microbial community dynamics. By leveraging ARM-mediated quorum sensing and its modular molecular architecture, researchers are developing innovative strategies to combat antibiotic-resistant infections, modulate microbiomes, and enhance agricultural sustainability [31].

The increasing prevalence of multidrug-resistant (MDR) bacteria has reignited interest in phage therapy as a viable alternative to traditional antibiotics [33]. Through gene-editing techniques, scientists have engineered phages with expanded host ranges and enhanced efficacy against pathogenic bacteria. For instance, modifications to receptor-binding proteins have enabled phages to target a broader spectrum of bacterial strains, addressing the challenge of bacterial resistance to phage infection.

Furthermore, the integration of CRISPR-Cas systems into phages has significantly improved their antibacterial potency [25]. Engineered phages equipped with CRISPR-Cas machinery can selectively target and degrade bacterial genomes, thereby minimizing the emergence of phage-tolerant bacterial populations. This approach has demonstrated promising results in preclinical studies, effectively eliminating biofilm-associated bacteria and outperforming wild-type phages in competitive environments [32,34].

Beyond direct antibacterial applications, phages are being harnessed to precisely modulate microbial communities [25]. Due to their inherent specificity, phages can selectively alter microbiome composition without disrupting beneficial microbes. For example, targeted phage interventions have been employed to reduce specific bacterial populations within complex microbiomes, thereby restoring microbial balance in health- and disease-associated ecosystems [26,35].

In agriculture, phages present an environmentally sustainable alternative to chemical antibiotics for controlling bacterial pathogens [40]. Phage-based interventions have been shown to reduce the prevalence of harmful bacteria in crops and livestock, thereby promoting plant and animal health while mitigating the spread of antibiotic resistance. This strategy aligns with sustainable agricultural practices by minimizing chemical inputs and preserving beneficial microbiota [41,42,43].

Advancements in synthetic biology and high-throughput screening are poised to further enhance phage engineering [44]. Techniques such as CRISPR-Cas-mediated genome editing and directed evolution are being employed to develop phages with tailored traits, including expanded host ranges and enhanced lytic capabilities [25]. Ongoing research aims to refine these methodologies to create phage therapies that are both safe and effective for clinical and environmental applications [31].

ARM systems provide a programmable platform for regulating phage lifecycles with high precision [45]. However, it should be noted that most ARM modules have so far been identified in temperate phages, which are traditionally regarded as less suitable for therapeutic applications because of their lysogenic potential [46]. Therefore, instead of directly “revolutionizing” phage therapy, the more realistic near-term promise of ARM lies in serving as a design principle for controllable infection dynamics [47]. Such principles could be leveraged by transferring ARM-like modules into strictly lytic phages, or by integrating them into synthetic biology frameworks for applications such as microbiome engineering, programmable antimicrobial interventions, and community-level regulation. In this way, ARM systems may complement rather than replace existing phage therapy strategies, offering new avenues for precision and adaptability in antimicrobial design [48,49]. However, realizing this promise requires addressing technical challenges, advancing synthetic biology tools, and establishing ethical guidelines. As research progresses, the most promising direction is to integrate ARM modules into broader synthetic biology frameworks, where controllable lifecycle decisions could be combined with programmable sensing circuits, targeted delivery platforms, or community-level interventions [50]. Realizing these possibilities will require advances in genome engineering, predictive modeling, and ethical guidelines. The next step, therefore, is not only to refine technical feasibility but also to define realistic scenarios where ARM-inspired design principles can complement existing phage therapies and microbiome interventions.

## 6. Conclusions

ARM systems represent a crucial regulatory mechanism in phage biology, governing bacterial population dynamics, horizontal gene transfer, and microbial ecosystem stability [2]. Their ability to modulate the lytic–lysogenic switch provides a foundation for optimizing phage therapy and microbiome engineering. Recent studies have underscored the versatility of ARM systems across diverse phage species and their potential applications in clinical, agricultural, and environmental contexts. As research advances, harnessing these systems could drive innovative solutions for antimicrobial resistance, ecosystem management, and precision microbiome interventions [37].

Future research should prioritize the exploration of ARM system diversity, their evolutionary trajectories, and their translational potential. A comprehensive understanding of ARM system variability and its underlying molecular mechanisms will be essential for developing more effective and targeted phage therapies [51]. Additionally, advancements in synthetic biology present exciting opportunities to engineer ARM systems for precise control over phage lifecycle decisions, paving the way for innovative strategies to combat antibiotic-resistant infections and enhance microbial ecosystem stability [22,52]. Through a deeper understanding of phage communication networks, the ARM system may provide substantial potential for advancing future studies in microbial management and the mitigation of antibiotic resistance.

## Figures and Tables

**Table 1:** Comparative analysis of key features between the bacteriophage Arbitrium (ARM) communication system and the classic lambda phage CI/Cro regulatory system.

Comparative Dimension	ARM System	Lambda Phage CI/Cro System	The Uniqueness of the ARM System	Ref.
Signal Type	Small peptide (AimP)	Proteins (CI and Cro)	The first peptide-mediated quorum sensing mechanism	[2,4]
Environmental Response	Directly sensing host density	Dependent on host stress signals (e.g., SOS response)	Dynamically adapts to population pressure, reducing host dependence	[5,6]
Regulatory Architecture	Conformational changes in the TPR domain inhibit DNA binding	Competitive binding of HTH proteins to operator sites	Eukaryote-like signaling mechanism with high modularity	[4,7,9]
Therapeutic Applications	Easily engineered control of the lysis/lysogeny balance	Complex to modify, with efficacy easily affected by host state	Precise and controllable, suitable for customized phage therapy	[10,24]

## Data Availability

No new data were created or analyzed in this study. Data sharing is not applicable to this article.
